# RFDAF-Net: a novel region-specific feature decoupling and adaptive fusion network for field soybean disease identification in precision agriculture

**DOI:** 10.3389/fpls.2025.1734292

**Published:** 2026-01-15

**Authors:** Renyong Pan, Qihang Yang, Yang Chen, Jian Cao

**Affiliations:** 1 College of Microelectronics and Artificial Intelligence, Kaili University, Kaili, China; 2 School of Computer and Information, Qiannan Normal University for Nationalities, Duyun, China; 3 College of Big Data and Information Engineering, Guizhou University, Guiyang, China; 4 Micronano and Intelligent Manufacturing Engineering Research Centre of Ministry of Education, Kaili, China

**Keywords:** adaptive fusion, deep learning, precision agriculture, region-specific feature decoupling, soybean disease identification

## Abstract

**Introduction:**

Soybean diseases pose a significant threat to global crop yield and food security, necessitating rapid and accurate identification for effective management. While deep learning offers promising solutions for plant disease recognition, existing models often struggle with the complexities of in-field soybean disease identification, particularly due to high intra-class variations and subtle inter-class differences.

**Methods:**

To address these challenges, we propose a novel region-specific feature decoupling and adaptive fusion network (RFDAF-Net) designed for robust and precise soybean disease recognition under real-world field conditions. The core of RFDAF-Net consists of two key components: a region-specific feature decoupling (RFD) module that enhances discriminative patterns and suppresses redundant information through a dual-pathway design, explicitly separating shallow, intermediate, and deep features; and a region-specific feature adaptive fusion (RFAF) module that dynamically integrates these multi-scale features via learned spatial attention. This hierarchical feature decomposition effectively isolates discriminative disease signatures while suppressing irrelevant variations. The architecture is flexible, enabling seamless integration with various backbone networks including both convolutional neural networks and Transformers.

**Results:**

We evaluate RFDAF-Net extensively on a comprehensive soybean disease dataset containing images captured in diverse field environments. Experimental results show that our method significantly outperforms current state-of-the-art models across multiple architectures, achieving a top accuracy of 99.43% when implemented with a Swin-B backbone.

**Discussion:**

The proposed framework offers an interpretable and field-ready solution for precision crop protection, demonstrating strong generalization ability and practical utility for real-world agricultural applications.

## 1 Introduction

Soybean cultivation underpins global food systems as a primary source of plant-based proteins and edible oils, with production stability directly impacting agricultural economies worldwide (Dilawari et al., 2022). Climate-induced environmental volatility, particularly erratic precipitation and rising temperatures, has accelerated the spread of destructive diseases including soybean rust, frogeye leaf spot, and sudden death syndrome (Hossain et al., 2024). Field diagnosis faces persistent challenges due to overlapping visual symptoms during early infection stages, limited access to expert pathology services in rural areas, and phenotypic plasticity under varying field conditions (Lin et al., 2025; Sharma et al., 2025). These constraints often trigger indiscriminate fungicide applications, escalating production costs while generating ecological risks through chemical runoff and non-target organism harm. Consequently, automated disease recognition systems have become critical for enabling precision interventions that reduce pesticide dependency while supporting sustainable soybean production (Pranta et al., 2025).

To address these urgent needs, computer vision-based approaches have become the cornerstone of modern plant phenotyping and disease diagnosis (Upadhyay et al., 2025). The field has undergone a significant paradigm shift, moving from traditional machine learning methods that relied on hand-crafted feature extraction (e.g., color, texture, and shape histograms) to the end-to-end feature learning capabilities of deep learning (DL) (Rumpf et al., 2010; Hari and Singh, 2025). This transition has established DL as the predominant methodology for in-field applications, owing to its superior ability to automatically learn discriminative and hierarchical features from raw image data. Among various DL architectures, convolutional neural networks (CNNs) have been extensively adopted as the backbone for plant disease recognition (Parez et al., 2025; Chakrabarty et al., 2024). Pioneering and widely used architectures such as ResNet (He et al., 2016), Inception (Szegedy et al., 2015), and EfficientNet Tan and Le (2019) have demonstrated remarkable accuracy in classifying crop diseases, effectively learning to distinguish subtle patterns indicative of pathological stress. More recently, Visual Transformers (VTs) have emerged as a powerful alternative, leveraging self-attention mechanisms to capture global contextual relationships within an image, often achieving state-of-the-art performance. However, despite their demonstrated prowess (Dosovitskiy, 2020; Liu et al., 2021), both CNN and VT architectures often exhibit suboptimal performance when deployed against the inherent complexities of real-world field-based plant disease diagnosis (Kumar et al., 2023). A significant limiting factor is that these naive networks struggle to capture fine-grained pathological features with high fidelity. The common strategy of merely leveraging deeper hierarchical representations proves insufficient for encapsulating the complete semantic information of a disease, as critical early-stage symptomatic details can be lost or diluted through successive pooling and non-linear transformations (Lin et al., 2025). While multi-scale feature fusion frameworks, such as feature pyramid networks (FPN) (Lin et al., 2017), have been proposed to mitigate this issue by combining features from different depths, they often inadvertently learn redundant feature patterns across scales. This redundancy limits their effectiveness, as simply aggregating features without explicit guidance does not necessarily ensure that each scale contributes distinct and complementary information. The fundamental challenge, therefore, lies not only in extracting multi-scale features but in explicitly enabling each level to learn discriminative and non-repetitive feature patterns. An ideal solution would orchestrate the feature extraction process such that shallower layers, with their higher spatial resolution, are sharpened to focus on low-level textural patterns, color variances, and localized edge information indicative of early infection. Concurrently, deeper layers should be refined to excel at integrating these details into a robust high-level semantic understanding of the disease phenotype. How to disentangle and maximize the utility of these heterogeneous representations across scales remains a pivotal and unresolved research question.

To address the challenges of feature redundancy and insufficient feature granularity in multi-scale learning, we propose a novel region-specific feature decoupling and adaptive fusion network (RFDAF-Net) for robust soybean disease recognition in complex field environments. Distinct from previous approaches, our model introduces two mechanism-specific innovations. First, unlike traditional attention mechanisms (Hu et al., 2018) that solely focus on highlighting salient regions, we design a region-specific feature decoupling (RFD) module equipped with a dual-branch strategy of simultaneous enhancement and suppression. While standard attention tends to converge on the most obvious discriminative parts, the RFD module explicitly suppresses these activated regions in a parallel branch. This mechanism forces the network to decouple features and mine complementary visual cues from non-dominant regions, thereby enriching the feature diversity across varying depths. Second, to overcome the limitations of naive multi-scale fusion methods, we introduce a region-specific feature adaptive fusion (RFAF) module. Instead of treating features from shallow, intermediate, and deep layers equally (Lin et al., 2017), the RFAF module employs a content-aware gated integration mechanism. It dynamically computes spatial weight maps for each scale, allowing the network to adaptively filter out noise from shallow layers while selectively retaining high-level semantic concepts from deep layers based on the specific input context.

The main contributions of this work are summarized as follows:

1. We propose a novel region-specific feature decoupling and adaptive fusion network (RFDAF-Net) for identification of soybean diseases. RFDAF-Net explicitly decouples and adaptively reintegrates multi-scale features to enhance the extraction of discriminative patterns while suppressing redundancy, effectively improving feature representativeness.
2. The proposed architecture demonstrates broad compatibility with both CNN and Vision Transformer backbones, consistently enhancing soybean disease recognition performance across diverse network frameworks.
3. We establish a state-of-the-art performance for in-field soybean disease recognition on a challenging dataset, demonstrating substantial improvements in accuracy over strong baseline models.

## 2 Related work

The application of computer vision and deep learning for plant disease recognition has evolved significantly, progressing from laboratory settings towards in-field settings (Antwi et al., 2024; Tian et al., 2024). This section reviews the relevant literature in three key areas: traditional image processing-based methods, the rise of deep learning-based approaches, and recent advancements in addressing the challenges of agricultural environments.

Traditional Image Processing-Based Methods: Rumpf et al. (2010) proposed an early detection method for sugar beet diseases using support vector machine (SVM) with spectral vegetation indices, achieving up to 97% accuracy in distinguishing diseased leaves and demonstrating potential for presymptomatic disease identification. Prasad et al. (2012) developed a plant biometric system using Gabor wavelet transform (GWT) and SVM for crop disease detection, achieving robust accuracy of approximately 89% across various conditions. The method aids in guiding farmers on disease control to improve production. Wang et al. (2012) developed a plant disease recognition system using K-means segmentation and BP neural networks with multiple visual features, achieving 100% accuracy in identifying grape and wheat diseases. However, these traditional approaches often depend on manually designed features and shallow classifiers, which struggle to capture complex and hierarchical disease characteristics, leading to limited adaptability and generalization across diverse disease appearances.

Deep Learning for Plant Disease Recognition: Atila et al. (2021) employed EfficientNet architectures (B4 and B5) with transfer learning for plant disease classification on the PlantVillage dataset, achieving superior accuracy up to 99.97% and precision up to 99.39%, significantly outperforming other deep learning models. Macdonald et al. (2024) proposed a novel lightweight CNN with dense residual connections for plant disease classification without pre-training, achieving high performance (96.75% accuracy, 97.62% precision) with only 228K parameters, demonstrating computational efficiency comparable to larger models on the PlantVillage dataset. Albahli (2025) proposed a lightweight EfficientNetV2-based model integrating RGB, multispectral drone imagery, and IoT sensor data, achieving 94.3% accuracy with 28.5 ms inference time and a 30% parameter reduction, demonstrating strong efficiency and robustness for edge-deployable crop disease diagnosis. While existing methods achieve high accuracy in controlled settings, their performance often degrades in real field conditions due to complex backgrounds, variable lighting, and occlusions. This highlights the critical need for models specifically designed to address the challenges of in-field plant disease recognition.

Challenges in Field-Based Diagnosis: Liu et al. (2024) proposed a multi-scale deformable convolution network for apple leaf disease detection, utilizing dual-branch convolution and constrained offset intervals to handle varying lesion scales and deformable geometries, achieving 66.8% accuracy in complex natural environments. Li et al. (2024) applied transfer learning with a frozen Xception backbone to identify 10 tea pests and diseases in complex plantation environments, achieving 98.58% test accuracy without attention mechanisms, demonstrating high practical utility for Yunnan tea cultivation. Huang et al. (2025) proposed a hybrid ConvNeXt-ViT model with ECA and DropKey for robust apple disease recognition, achieving 99.2% accuracy in lab conditions and 79.3% in natural environments, outperforming ViT/ConvNeXt/ResNet50 by 18.6–37.8% in field generalization. While existing deep learning models have laid a solid foundation for automated field plant disease diagnosis, their direct application to complex in-field soybean disease recognition remains constrained. Current architectures still struggle to fully address challenges such as fine-grained feature loss, redundant multi-scale representations, and sensitivity to field noise. In particular, the limitations of existing feature fusion strategies motivate the need for a more sophisticated approach to explicitly disentangle and adaptively integrate feature representations.

## 3 Materials and methods

### 3.1 Data collection and pre-processing

**Figure 1:**
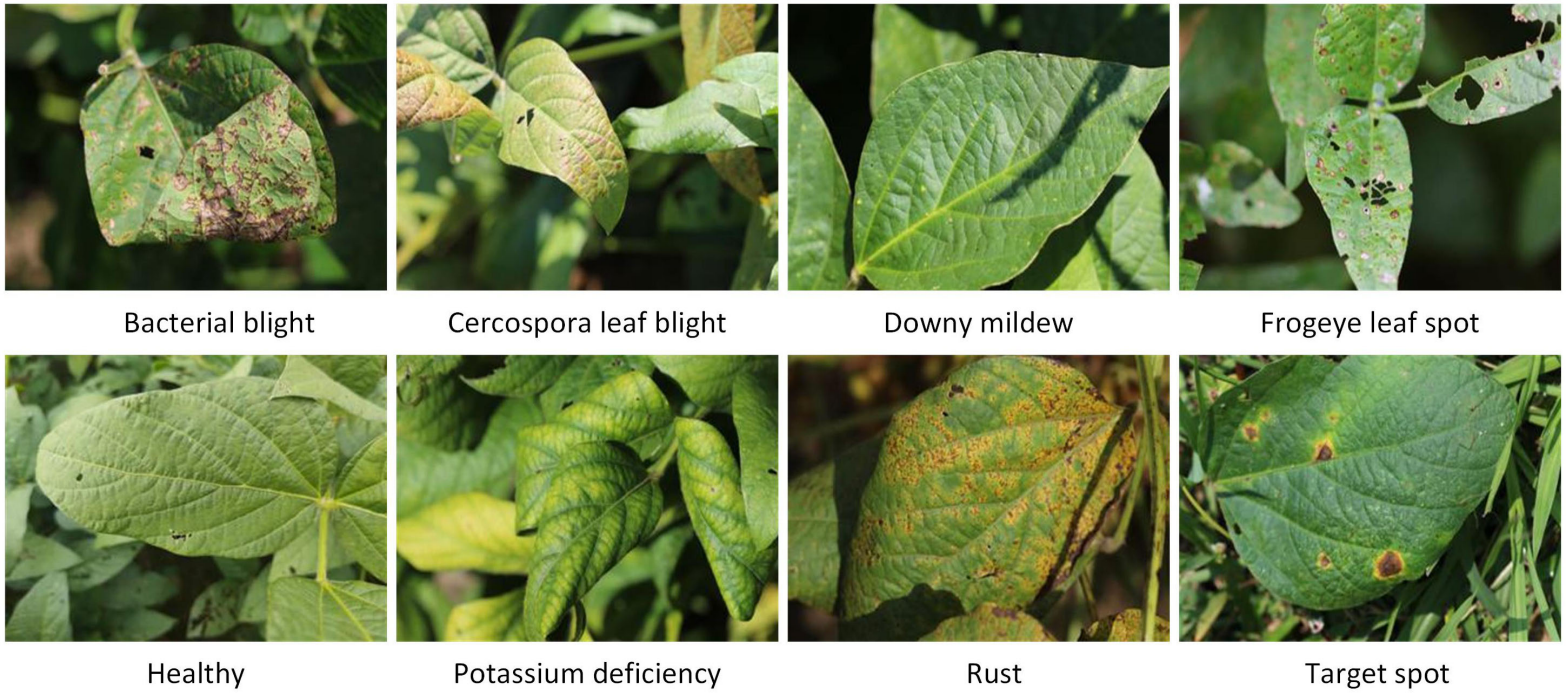
Representative samples of each category.

All experimental data employed in this research were obtained from the publicly accessible auburn soybean leaf disease image dataset (Bevers et al., 2022). This dataset comprises soybean leaf images captured under real field conditions across multiple growing seasons (2020–2021) in the United States, using both smartphone cameras and digital single-lens reflex (DSLR) cameras to ensure diversity in imaging quality and perspective. It encompasses eight categories of soybean leaf conditions: Bacterial blight, Cercospora leaf blight, Downy mildew, Frogeye leaf spot, Healthy, Potassium deficiency, Soybean rust, and Target spot. The images exhibit significant variation in background contexts, including complex field environments, uniform white backgrounds, and grassy settings, which enhances the robustness and generalizability of models trained on this data. For the purposes of this study, a total of 9,648 images were selected from ASDID after initial screening for quality and label consistency. Example images from each category are provided in **Figure 1**.

Considering the varied and often high resolutions of images within the dataset, as well as the imbalanced distribution across disease categories, preprocessing is essential prior to model training to improve computational efficiency and stabilize learning. To address this, all soybean leaf images were resized to a uniform dimension of 224×224 pixels. The dataset was then partitioned into training, validation, and test sets with a ratio of 7:1:2 per category, ensuring representative sampling across all classes. The training set is used to optimize model parameters through backward propagation, while the validation set enables hyperparameter tuning and early stopping to prevent overfitting. The test set, kept entirely separate and unused in any training phase, provides an unbiased evaluation of the final model’s generalization performance on unseen data. In addition, deep learning models typically require large volumes of training data to achieve robust generalization and mitigate overfitting. To augment the dataset, we applied a series of geometric and pixel-level transformations. These included random rotations, horizontal and vertical flipping, color jittering, brightness adjustment, additive Gaussian noise, and contrast limited adaptive histogram equalization (CLAHE). The data distributions before and after augmentation are shown in **Table 1**. Such augmentations simulate imaging variations under real-world conditions and enhance the model’s ability to recognize disease patterns under diverse environments.

**Table 1:** Data distributions before and after augmentation.

Classes	Before augmentation				After augmentation		
	Train	Val	Test	Total	Train	Val	Test
Bacterial blight	339	48	97	484	2373	48	97
Cercospora leaf blight	1119	159	320	1598	2238	159	320
Downy mildew	457	65	130	652	2285	65	130
Frogeye leaf spot	1078	154	308	1540	2156	154	308
Healthy	1143	163	326	1632	2286	163	326
Potassium deficiency	724	103	207	1034	2172	103	207
Soybean rust	1139	162	326	1627	2278	162	326
Target spot	757	108	216	1081	2271	108	216
Total	6756	962	1930	9648	18059	962	1930

### 3.2 The proposed RFDAF-Net

**Figure 2:**
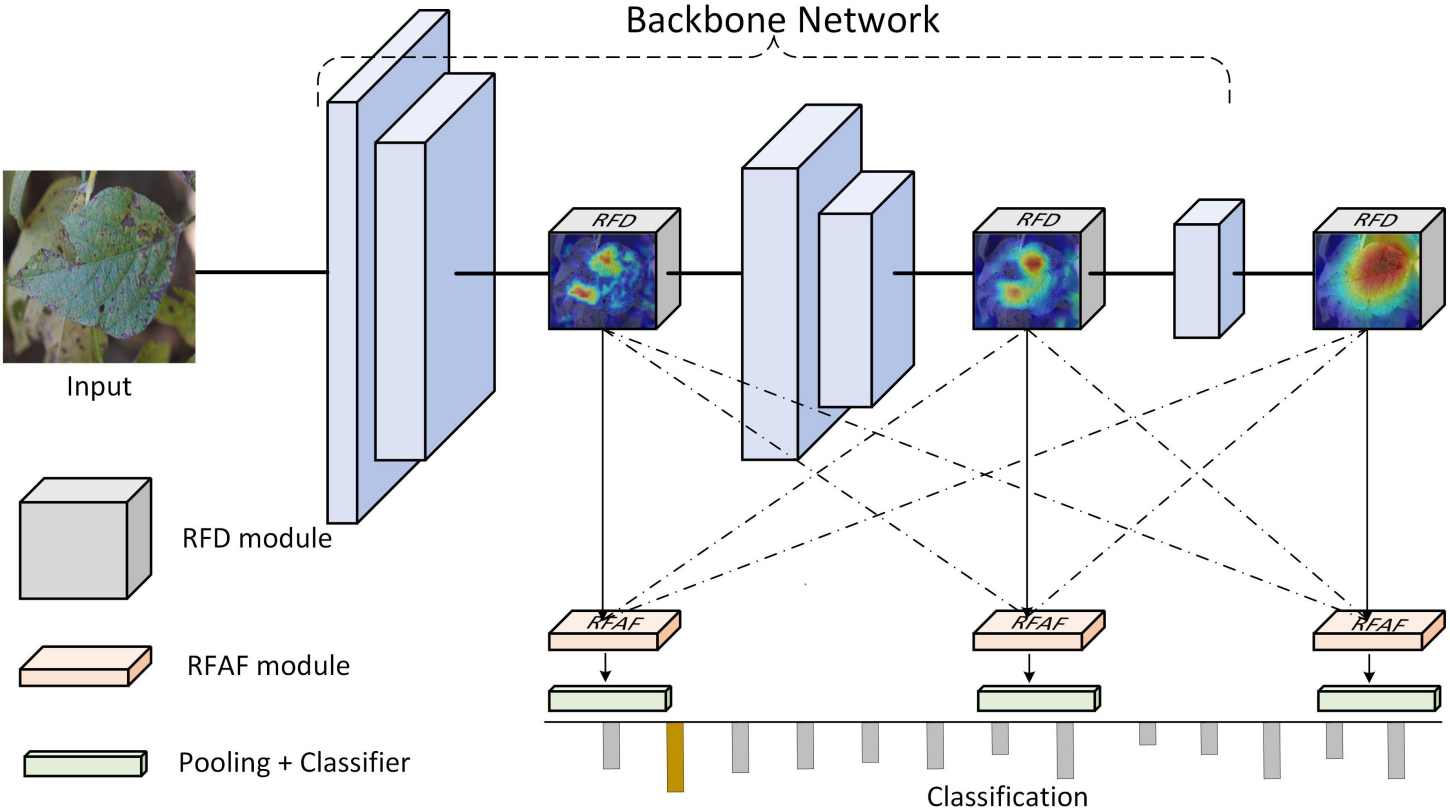
The overall framework of the proposed RFDAF-Net. It consists of the backbone network, the RFAF modules, and the RFAF modules.

The overall architecture of the proposed RFDAF-Net is illustrated in **Figure 2**. The model consists of two core components: a region-specific feature decoupling (RFD) module and a region-specific feature adaptive fusion (RFAF) module, which can be flexibly integrated into various backbone networks such as ResNet or Swin Transformer. The RFD module is designed to enhance discriminative features and suppress redundant information through a dual-pathway structure, enabling explicit separation of fine-grained details in shallow layers and high-level semantics in deeper layers. The RFAF module dynamically fuses these multi-scale disentangled features using a content-aware spatial weighting mechanism to achieve adaptive feature integration. Specifically, an input image is first processed by the backbone network to generate multi-level feature maps. These features are then fed into the RFD module, where low-level features are refined to retain detailed textural and lesion information, while high-level features are purified to emphasize semantic concepts. The disentangled features from different levels are subsequently merged by the RFAF module, which assigns spatially varying weights to emphasize informative regions and suppress noise. The fused feature representation is finally passed to a classifier to produce the disease probability distribution.

### 3.3 Region-specific feature decoupling module

**Figure 3:**
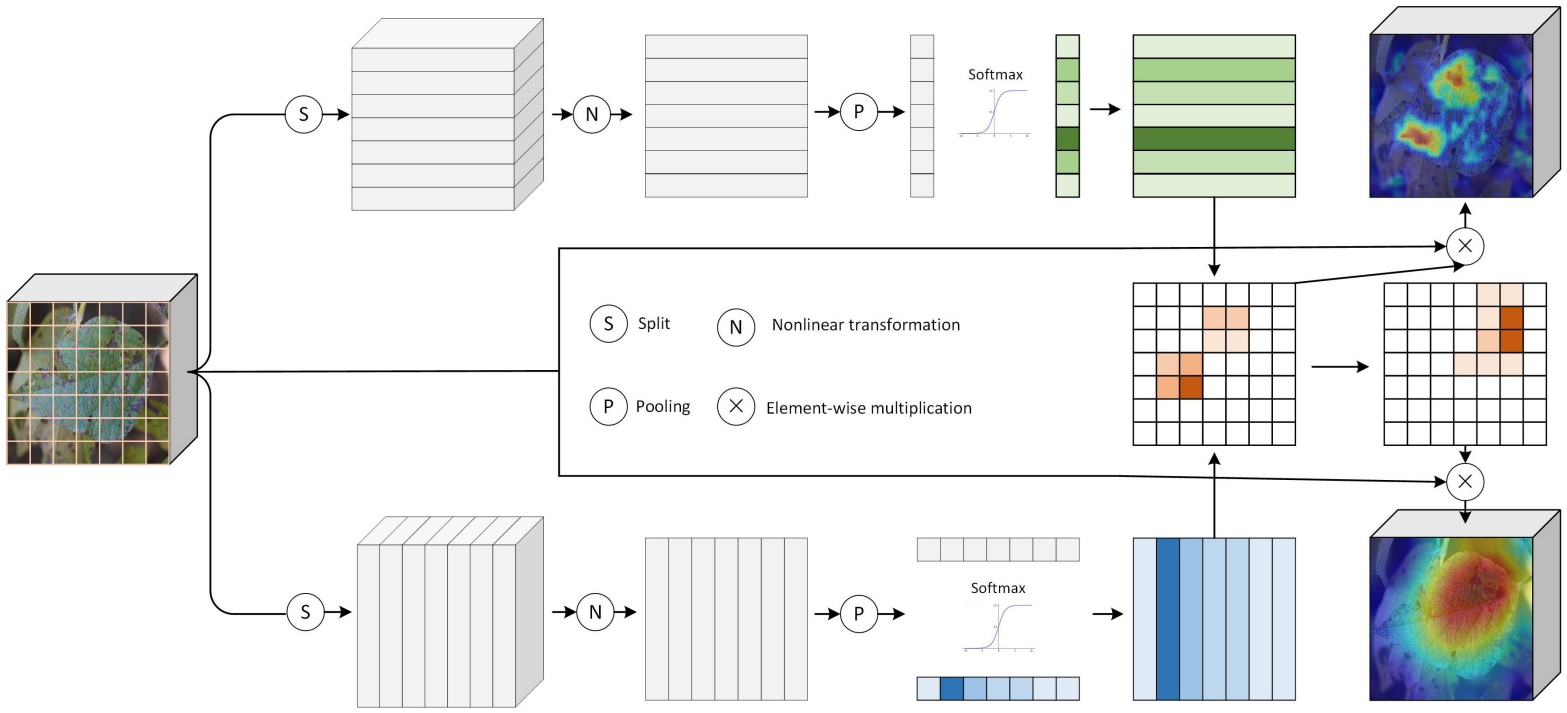
The RFD module.

Current deep learning models applied to soybean disease recognition under field conditions predominantly rely on features from a single network depth for classification. While these features provide a broad receptive field, they often fail to capture sufficiently discriminative details under conditions of high visual similarity, such as those commonly encountered in fine-grained soybean disease identification. These challenges are compounded by low inter-class variance among different diseases and high intra-class variance due to varying symptom manifestations across growth stages and environmental conditions. To mitigate these issues, we introduce a region-specific feature decoupling (RFD) module. This module is designed to explicitly extract hierarchical and complementary information by emphasizing salient regions while suppressing less informative areas. The structure of the RFD module is shown in **Figure 3**. It employs a dual branch mechanism comprising a feature enhancement branch and a feature suppression branch. The enhancement branch amplifies semantically important regions in the current feature map, whereas the suppression branch attenuates the influence of those same regions in subsequent layers. This process encourages the network to identify new distinctive features in later stages. For example, regions showing high activation in shallow feature maps, which often correspond to early textural symptoms, are reinforced by the enhancement branch. At the same time, the suppression branch reduces the emphasis on these regions in deeper feature maps, directing the network’s attention to other discriminative parts of the image. This cross-scale interaction enables the model to learn diverse and complementary visual cues across different depths, significantly improving the discriminative power and robustness of the feature representations for accurate in field soybean disease recognition.

The region-specific feature decoupling (RFD) module processes an input tensor $X\in {\mathbb{R}}^{C\times W\times H}$, where *C* indicates the number of channels, *H* and *W* represent the height and width, respectively. The input is first partitioned into 7 segments along both the horizontal and vertical axes. Each horizontal segment is denoted as $X_{i}^{\left(w\right)}\in {\mathbb{R}}^{C\times (H/7)\times W}$ for *i* = 1, 2, …, 7, and each vertical segment as $X_{j}^{\left(h\right)}\in {\mathbb{R}}^{C\times H\times (W/7)}$ for *j* = 1, 2, …, 7.

Then, these two outputs undergo nonlinear transformations to yield $F_{i}^{\left(w\right)}$ and $F_{j}^{\left(h\right)}$, respectively. Note that both $F_{i}^{\left(w\right)}$ and $F_{j}^{\left(h\right)}$ have a channel number of 1. Next, these two tensors apply global average pooling (GAP) followed by softmax activation and broadcasting, thereby generating enhanced weights for each direction (Equations 1, 2):

(1) $$E_{w}=B\;[\text{Softmax}\;(\text{GAP}(F_{i}^{\left(w\right)}))]$$

(2) $$E_{h}=B\;[\text{Softmax}\;(\text{GAP}(F_{j}^{\left(h\right)}))]$$

Where *B*[·] represents the broadcasting. Here, $E_{w}=[e_{1}^{\left(w\right)},e_{2}^{\left(w\right)},\ldots ,e_{7}^{\left(w\right)}]$ and $E_{h}=[e_{1}^{\left(h\right)},e_{2}^{\left(h\right)},\ldots ,e_{7}^{\left(h\right)}]$ contain the enhancement coefficients for the horizontal and vertical directions, respectively. The enhanced feature map *Y_E_* is subsequently computed as (Equation 3):

(3) $$Y_{E}=X+(X⊗E_{w}⊗E_{h})$$

The suppression feature map *Y_S_* is defined as (Equation 4):

(4) $$Y_{S}=X⊗S_{w}⊗S_{h}$$

The suppression weights $s_{i}^{\left(w\right)}$ and $s_{j}^{\left(h\right)}$ are determined by (Equation 5):

$s_{i}^{\left(w\right)}=\{\begin{array}{ll} 1-\alpha , & \text{if}\;e_{i}^{\left(w\right)}=\max(E_{w})\\ 1, & \text{otherwise} \end{array},\;$ $s_{j}^{\left(h\right)}=\{\begin{array}{ll} 1-\alpha , & \text{if}\;e_{j}^{\left(h\right)}=\text{max}(E_{h})\\ 1, & \text{otherwise} \end{array}$

Where α is a hyperparameter and $S_{w}=[s_{1}^{\left(w\right)},\ldots ,s_{7}^{\left(w\right)}]$, $S_{h}=[s_{1}^{\left(h\right)},\ldots ,s_{7}^{\left(h\right)}]$.

### 3.4 Region-specific feature adaptive fusion module

While the RFD module effectively extracts multi-scale discriminative features, effectively integrating these hierarchical representations remains challenging. Most existing fusion methods simply concatenate or sum multi-level features, ignoring the distinct contributions of shallow, intermediate, and deep feature maps. This often leads to feature redundancy and limits the model’s ability to adaptively emphasize the most relevant information across spatial and semantic levels. To address this, we propose a Region-specific Feature Adaptive Fusion (RFAF) module, which dynamically combines features from multiple network depths through a learned weighting mechanism. The RFAF module consists of three dedicated branches processing shallow, intermediate, and deep feature maps, respectively. The shallow branch preserves fine-grained details such as texture and local lesions, the intermediate branch captures transitional patterns, and the deep branch encapsulates high-level semantic information.

**Figure 4:**
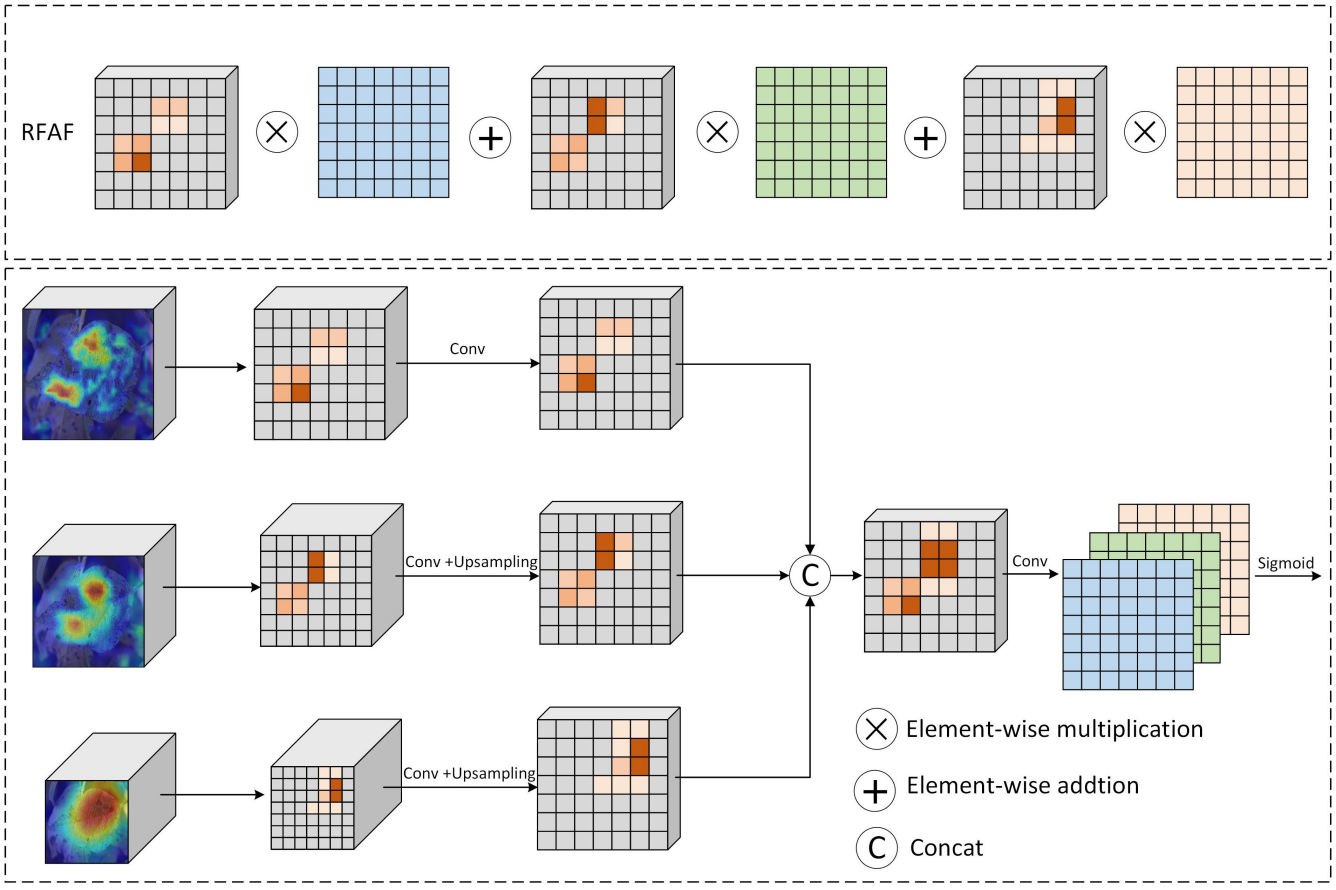
The RFAF module.

The core of the RFAF module lies in its adaptive fusion strategy. Feature maps from each branch are first aligned to a common spatial scale using up-sampling or down-sampling operations. These aligned features are then concatenated and processed through a convolutional layer to generate a compact representation. A sigmoid activation is applied to produce three adaptive weight maps corresponding to each branch. The final output is formed by a weighted summation of the original features using these learned weights, enabling the fusion process to emphasize the most informative features from each level in a context-aware manner. This design allows the RFAF module to effectively integrate complementary information while suppressing less relevant features. By dynamically adjusting the importance of features at different depths, the module significantly enhances the representational capacity of the network for improved soybean disease recognition under challenging field conditions. The structure of the RFAF module is illustrated in **Figure 4**.

The RFAF module synthesizes multi-scale feature representations through a gated integration mechanism. Let $F^{\left(1\right)}\in {\mathbb{R}}^{C_{1}\times H_{1}\times W_{1}}$, $F^{\left(2\right)}\in {\mathbb{R}}^{C_{2}\times H_{2}\times W_{2}}$, and $F^{\left(3\right)}\in {\mathbb{R}}^{C_{3}\times H_{3}\times W_{3}}$ denote the input feature tensors from the shallow, intermediate, and deep branches, respectively, each capturing distinct levels of semantic abstraction.

To facilitate cross-branch integration, we first apply feature transformation and spatial alignment. Features from branches 2 and 3 are processed through pointwise convolutional layers to project them into a common embedding space with reduced channel dimensionality (Equation 6):

(6) $$G^{\left(k\right)}=\phi (W_{k}*F^{\left(k\right)}+b_{k}),\;k=2,\;3$$

where $W_{k}$ and $b_{k}$ are learnable parameters of the 1×1 convolutions, and ϕ denotes the ReLU activation function. The transformed features $G^{\left(2\right)}$ and $G^{\left(3\right)}$ are then resized to the spatial dimensions of $F^{\left(1\right)}$ via bilinear interpolation, denoted as ${\tilde{G}}^{\left(2\right)}$ and ${\tilde{G}}^{\left(3\right)}$. The aligned features are concatenated along the channel dimension to form a composite representation (Equation 7):

(7) $$H=\text{Concat }(F^{\left(1\right)},{\tilde{G}}^{\left(2\right)},{\tilde{G}}^{\left(3\right)})$$

We then compute a set of spatially adaptive weight matrices through a fusion gate implemented as a 1×1 convolution followed by sigmoid activation (Equation 8):

(8) $$A=\sigma (W_{a}\;*\;H+b_{a})$$

where $A=[A_{1},A_{2},A_{3}]\in {\mathbb{R}}^{3\times H_{1}\times W_{1}}$ represents the attention weights for each branch. The final output is obtained via a weighted fusion (Equation 9):

(9) $$F_{\text{out}}=A_{1}⊗F^{\left(1\right)}+A_{2}⊗{\tilde{G}}^{\left(2\right)}+A_{3}⊗{\tilde{G}}^{\left(3\right)}$$

This formulation enables context-aware recombination of multi-scale features, enhancing discriminability while preserving structural details essential for accurate fine-grained recognition under challenging field conditions.

## 4 Experiments

### 4.1 Experimental setup and evaluation metrics

To ensure the reproducibility of our results, we detail the implementation and data settings as follows. The experiments were conducted on a workstation equipped with NVIDIA GeForce RTX 5070 Ti GPU and an Intel Core i5-12600KF CPU. The software environment was configured with Python 3.8 and PyTorch 1.13 on Windows 11. Regarding data settings and preprocessing, all input images were resized to a uniform dimension of 224 × 224 pixels using bilinear interpolation. We applied standard Z-score normalization using the ImageNet mean (*μ* = [0.485, 0.456, 0.406]) and standard deviation (*σ* = [0.229, 0.224, 0.225]). Data augmentation techniques, including random rotation, random flipping, and color jittering, were applied exclusively to the training set to enhance generalization, while the validation and test sets remained unaugmented to ensure unbiased evaluation. For the optimization procedure, we utilized the Stochastic Gradient Descent (SGD) optimizer with a momentum of 0.9 and a weight decay of 5×10^−4^. The batch size was set to 32. The model was trained for a total of 100 epochs to guarantee convergence. We employed a differential learning rate strategy: the backbone network, initialized with ImageNet-1K pre-trained weights 4, was fine-tuned with a lower learning rate of 2 × 10^−4^, while the newly added RFD and RFAF modules were initialized randomly and trained with a higher learning rate of 2 × 10^−3^. The suppression threshold *α* was set to 0.5. A summary of the key experimental configurations is provided in **Table 2**. To ensure the reliability of the experimental results, each model configuration was executed five times with different random seeds. The results reported in this paper correspond to the model checkpoint that achieved the highest accuracy on the validation set.

**Table 2:** Experimental environment details.

Category	Configuration
GPU	NVIDIA GeForce RTX 5070 Ti
CPU	Intel i5-12600KF
OS	Windows 11
Python Version	3.8.18
PyTorch Version	1.13.1
Backbone LR	2 × 10^−4^
New Modules LR	2 × 10^−3^
Optimizer	SGD (Momentum: 0.9)
Weight Decay	5 × 10^−4^
Batch Size	32
Total Epochs	100
Threshold (*α*)	0.5

The performance of the proposed model was quantitatively evaluated using four standard classification metrics: Accuracy (Acc), Precision (Pre), Recall (Rec), and F1-score (F1). These metrics were derived from the confusion matrix, which records true positives (TP), true negatives (TN), false positives (FP), and false negatives (FN). Their mathematical definitions are as follows (Equations 10–13):

(10) $$\text{Accuracy}=\frac{TP+TN}{TP+TN+FP+FN}$$

(11) $$\text{Precision}=\frac{TP}{TP+FP}$$

(12) $$\text{Recall}=\frac{TP}{TP+FN}$$

(13) $$\text{F}1-\text{score}=2\times \frac{\text{Precision}\times \text{Recall}}{\text{Precision}+\text{Recall}}$$

In addition to these quantitative metrics, interpretability tools such as Grad-CAM and t-SNE were employed to visualize decision regions and feature distributions, further validating the model’s reliability and explanatory capacity.

### 4.2 Comparison with state-of-the-art models

To evaluate the effectiveness of the proposed RFDAF-Net, we compared its performance against multiple state-of-the-art classification models on the soybean disease recognition task. As summarized in **Table 3**, RFDAF-Net achieves the highest accuracy of 99.43%, outperforming all competing approaches. Among the CNN-based models, VGG16 attained the lowest accuracy at 94.25%, while more modern architectures such as ResNet50 and EfficientNet-B0 reached around 97%. Transformer-based backbones generally delivered stronger performance, with Swin-B achieving 98.24%. Recent specialized architectures including TFANet and DIEC-ViT further improved performance, reaching 98.18% and 99.02%, respectively. Our RFDAF-Net, built upon a Swin-B backbone and enhanced with region-specific feature disentanglement and adaptive fusion mechanisms, attained a top accuracy of 99.43%. This result demonstrates the efficacy of the proposed modules in capturing discriminative multi-scale features and effectively integrating hierarchical information under challenging field conditions. The consistent improvement over strong baselines confirms that RFDAF-Net offers a robust solution for fine-grained agricultural image recognition.

**Table 3:** Comparison results with state-of-the-art models.

Model	Acc (%)
VGG16 (Simonyan and Zisserman, 2014)	94.25
ResNet50 (He et al., 2016)	97.26
MobileNetV2 (Sandler et al., 2018)	97.15
ShuffleNetV2 (Ma et al., 2018)	96.12
EfficientNet-B0 (Tan and Le, 2019)	97.10
ConvNeXt-B (Liu et al., 2022)	97.98
PiT-B (Heo et al., 2021)	97.36
PVT-B (Wang et al., 2021)	97.05
ViT-B (Dosovitskiy, 2020)	97.41
Swin-B (Liu et al., 2021)	98.24
TFANet (Pan et al., 2023)	98.18
DIEC-ViT (Lin et al., 2025)	99.02
RFDAF-Net (Ours)	99.43

### 4.3 Impact of the backbone networks

**Figure 5:**
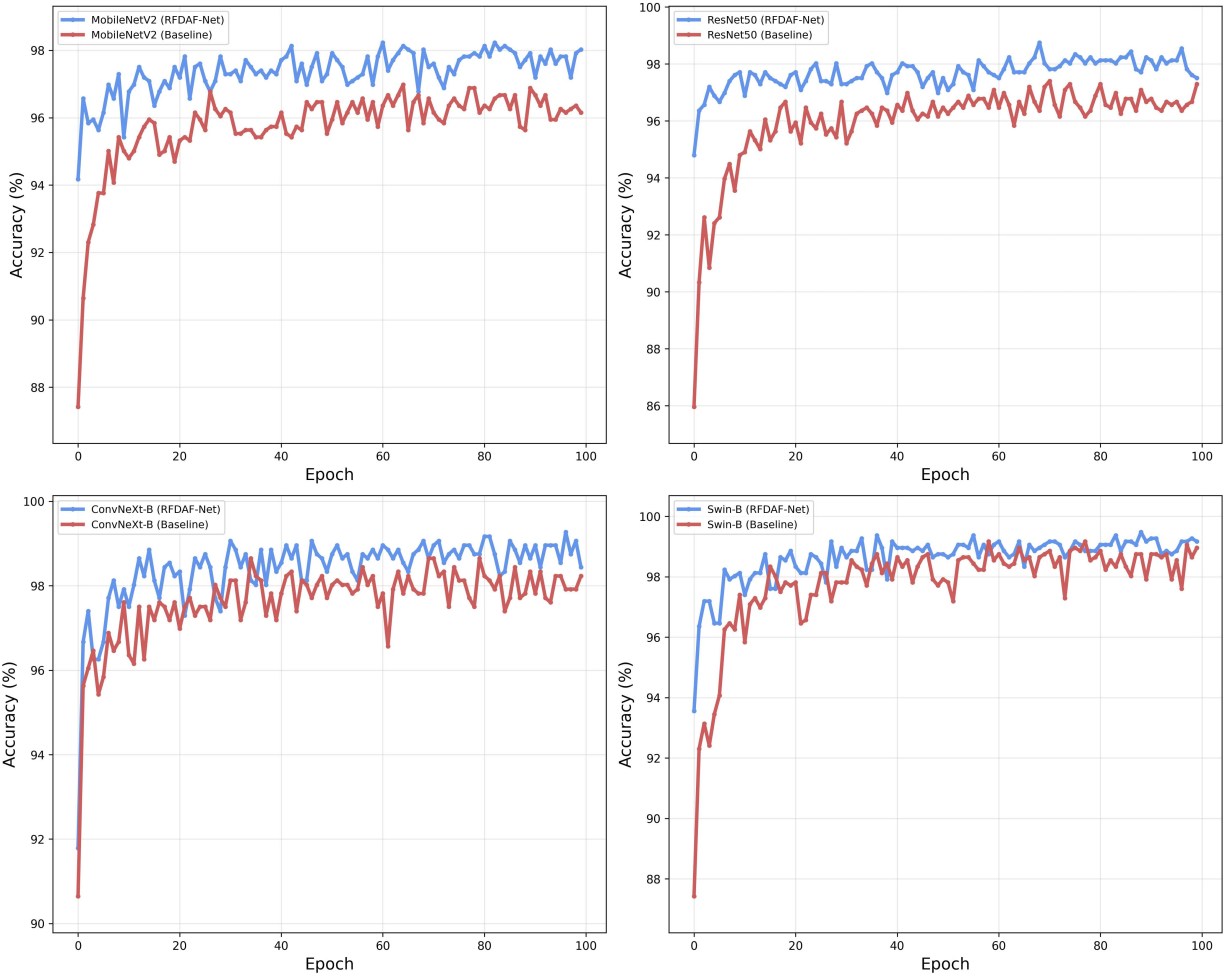
Impact of the different backbone networks on the validation set.

To evaluate the generalization ability of the proposed RFDAF-Net, we conducted extensive experiments using four different backbone networks: MobileNetV2, ResNet50, ConvNeXt-B, and Swin-B. The performance comparisons between the baseline backbones and their RFDAF-Net-enhanced variants on the validation set are illustrated in **Figure 5**. The results demonstrate that RFDAF-Net consistently improves classification accuracy across all backbone architectures. Specifically, when integrated with MobileNetV2, which served as a relatively weaker baseline, RFDAF-Net achieved the most significant performance gain. This suggests that the proposed feature disentanglement and adaptive fusion mechanisms effectively compensate for the limited representational capacity of lightweight backbones. With stronger backbones such as ResNet50 and ConvNeXt-B, RFDAF-Net still provided clear improvements, underscoring its ability to enhance even well-performing models. When combined with Swin-B, which already delivered high baseline accuracy, RFDAF-Net attained near-perfect performance. The marginal but consistent improvement in this case can be attributed to the fact that the classification task is approaching its performance ceiling, leaving limited room for further gains. These observations confirm that RFDAF-Net is robust and architecture-agnostic, providing measurable benefits across a diverse range of backbone networks. Its ability to achieve the largest improvements on weaker backbones is especially promising for practical applications where computational efficiency is critical. To further evaluate the robustness and generalization capability of RFDAF-Net, we compared its performance against baseline backbones on the test set. Quantitative results are presented in **Table 4**, which includes Accuracy, Precision, Recall, and F1-score for each model configuration. The results clearly demonstrate that RFDAF-Net consistently enhances performance across all backbone networks. Notably, the absolute performance of RFDAF-Net improves with the capacity of the backbone network—RFDAF-Net (Swin-B) achieves the highest results with 99.43% accuracy and 99.50% F1-score, while RFDAF-Net (MobileNetV2) also shows substantial gains, reaching 98.76% accuracy. This trend confirms that the effectiveness of our method is amplified when combined with more powerful feature extractors, yet it still brings significant improvements even on lighter backbones. Moreover, the conclusions drawn from the test set are fully consistent with those from the validation set: RFDAF-Net provides noticeable improvements across all architectures, with the most pronounced gains occurring on weaker backbones. The stable superiority under both validation and test environments strongly attests to the general applicability and robustness of the proposed method.

**Table 4:** Impact of the different backbone networks on the test set.

Model	Acc (%)	Pre (%)	Rec (%)	F1 (%)
MobileNetV2	97.15	97.47	97.20	97.33
ResNet50	97.26	97.41	97.32	97.36
ConvNeXt-B	97.98	98.04	97.93	97.96
Swin-B	98.24	98.60	97.65	98.09
RFDAF-Net (MobileNetV2)	98.76	98.87	98.98	98.92
RFDAF-Net (ResNet50)	98.96	98.94	99.02	98.98
RFDAF-Net (ConvNeXt-B)	99.28	99.38	99.28	99.32
RFDAF-Net (Swin-B)	99.43	99.53	99.48	99.50

### 4.4 Ablation analysis

To evaluate the individual contributions of the proposed components, we conducted ablation experiments using MobileNetV2 as the baseline backbone. The quantitative results are summarized in **Table 5**. The baseline MobileNetV2 achieves an accuracy of 97.15%. However, without explicit feature guidance, standard backbones often struggle to balance fine-grained textures with high-level semantics. Introducing the RFAF module alone improves accuracy to 97.92%. This improvement is not merely due to feature aggregation but stems from the RFAF’s gated attention mechanism. Unlike simple summation which propagates noise, RFAF dynamically assigns lower weights to irrelevant background clutter in shallow layers while amplifying semantic cues in deep layers, effectively ‘cleaning’ the representation before classification. Incorporating the RFD module yields a more substantial gain, reaching 98.18% accuracy. This performance leap validates the effectiveness of our region-specific decoupling strategy. By explicitly suppressing the most salient discriminative regions in the parallel branch, the RFD module forces the network to shift its attention to complementary visual evidence, such as subtle lesion margins or early-stage chlorosis patterns. This prevents the model from over-relying on a single dominant feature and enhances robustness against intra-class variations. Finally, combining both modules (RFDAF-Net) achieves the highest performance of 98.76%. This demonstrates a synergistic effect: the RFD module enriches the diversity of extracted features by mining non-salient patterns, while the RFAF module optimally integrates these diverse features by selectively emphasizing the most informative scales. Together, they form a closed-loop system that maximizes feature representativeness and discriminability.

**Table 5:** Ablation experiments on the MobileNetV2 backbone.

Backbone	RFD	RFAF	Acc (%)	Pre (%)	Rec (%)	F1 (%)
MobileNetV2			97.15	97.47	97.20	97.33
MobileNetV2	✓		98.18	98.24	98.55	98.38
MobileNetV2		✓	97.92	97.77	97.83	97.79
MobileNetV2	✓	✓	98.76	98.87	98.98	98.92

### 4.5 Visualization analysis

**Figure 6:**
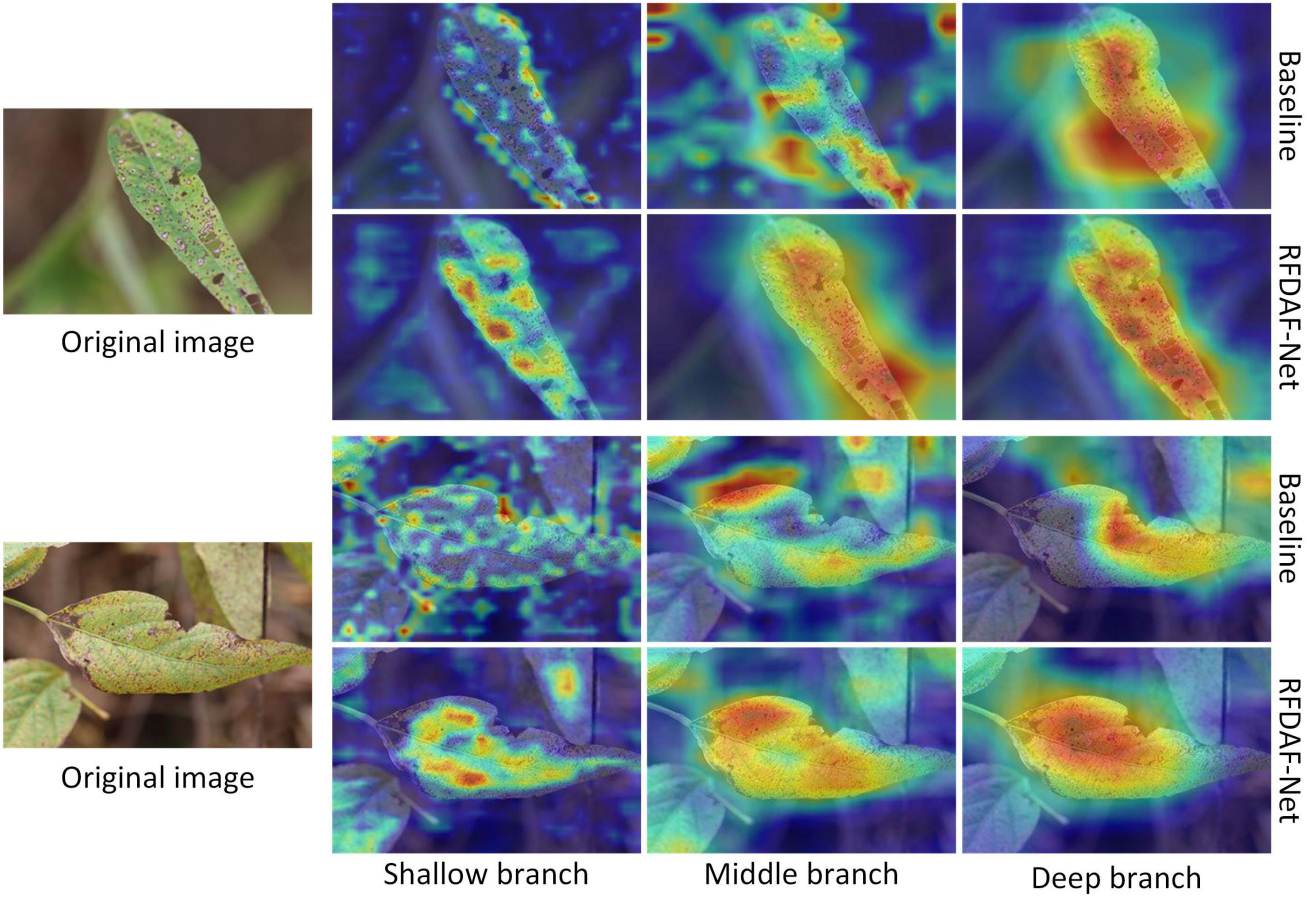
Grad-Cam visualization.

The Grad-CAM (Selvaraju et al., 2017) visualizations in **Figure 6** provide qualitative evidence of the effectiveness of the proposed RFDAF-Net module compared to a standard ResNet50 baseline. The visualizations reveal that RFDAF-Net produces more discriminative and semantically meaningful activation patterns across all feature levels. In the shallow branch, RFDAF-Net focuses sharply on fine-grained details such as lesion boundaries and textural variations, while the baseline model exhibits scattered and less interpretable activations. In the middle and deep branches, RFDAF-Net continues to maintain precise spatial localization of pathological regions, effectively capturing higher-level semantic features without losing resolution or contextual coherence. In contrast, the baseline model relies predominantly on its deep features for localization, with shallow and middle branches providing limited and often noisy contributions. These results demonstrate that RFDAF-Net enables each branch to specialize in capturing features at its respective scale, leading to more hierarchical and interpretable feature learning. The module’s ability to retain discriminative information across scales contributes significantly to its improved accuracy and robustness in complex field conditions.

**Figure 7:**
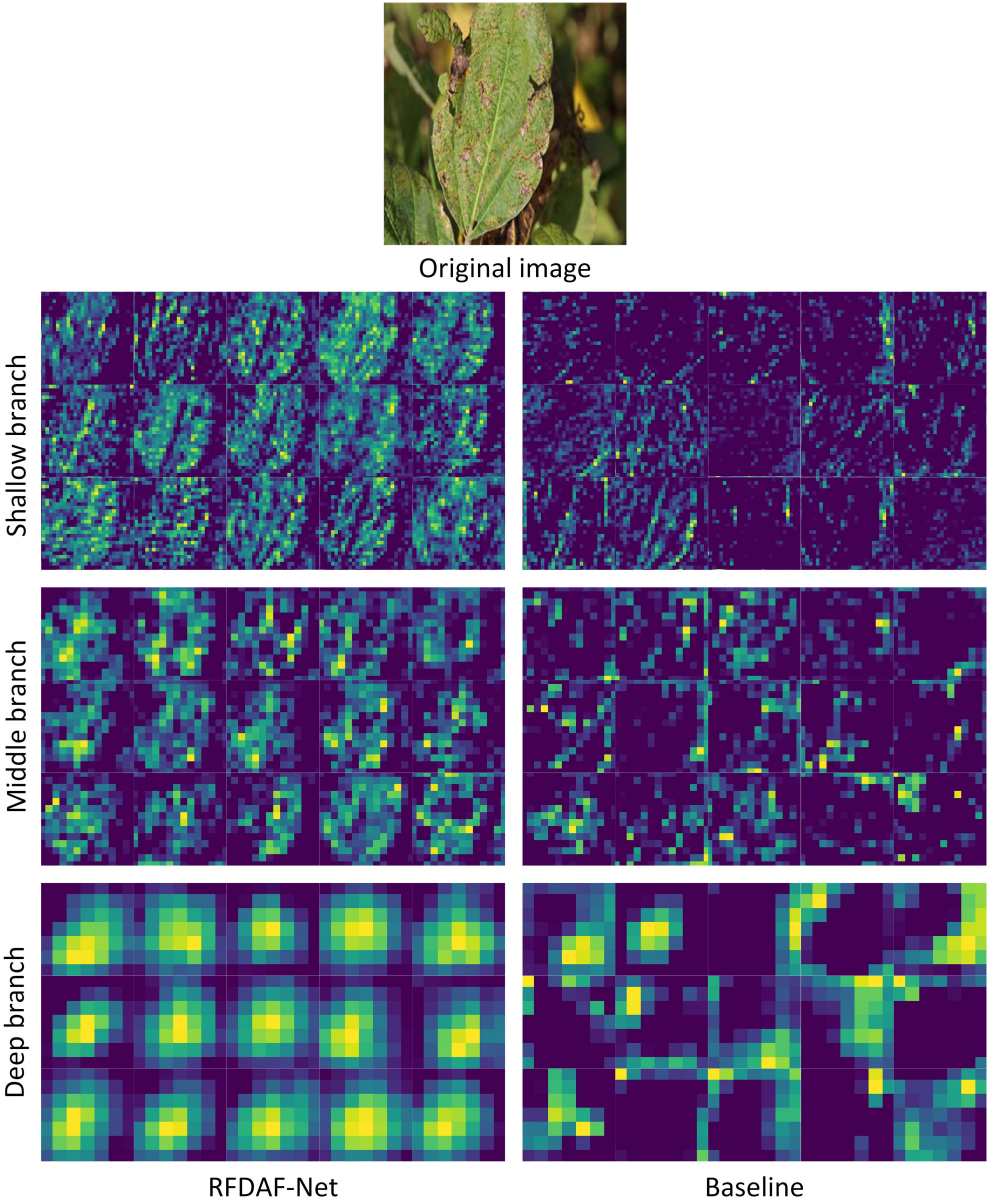
Feature map visualization.

**Figure 7** presents a comparative visualization of intermediate feature maps from both the baseline MobileNetV2 and the proposed RFDAF-Net across shallow, middle, and deep branches. The feature maps generated by RFDAF-Net exhibit stronger structural awareness and semantic coherence compared to those of the baseline. In the shallow layers, RFDAF-Net produces feature maps that clearly highlight fine-grained details such as edges, textures, and early symptomatic patterns. The middle-layer features show increased semantic abstraction while retaining spatial precision, effectively capturing transitional patterns indicative of disease progression. The deep features focus on high-level semantic concepts, such as the overall shape and extent of diseased regions, with minimal noise or irrelevant activations. In contrast, the baseline MobileNetV2 fails to maintain such hierarchical discriminability. Its shallow and middle features often appear noisy or semantically ambiguous, while the deep features, though somewhat consolidated, lack the spatial precision and interpretability of those produced by RFDAF-Net. These visual comparisons reinforce the quantitative results, confirming that RFDAF-Net enhances feature learning across all network levels, leading to more informative and task-relevant representations essential for accurate soybean disease recognition.

**Figure 8:**
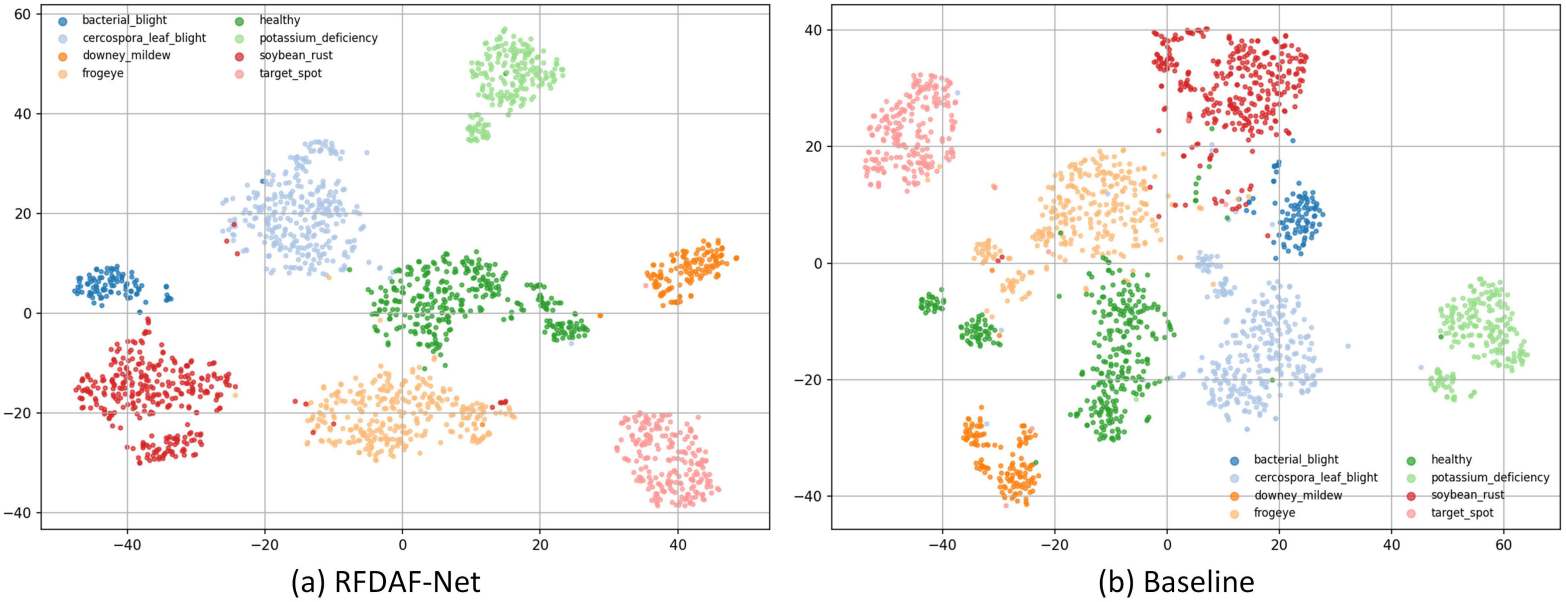
t-SNE visualization. **(a)** represents RFDAF-Net. **(b)** represents the Swin-B baseline.

The t-SNE (Maaten and Hinton, 2008) visualization in **Figure 8** illustrates the feature distribution of the baseline Swin-B model and the proposed RFDAF-Net in a two-dimensional embedded space. The feature representations generated by RFDAF-Net exhibit significantly improved class separability compared to those of the baseline. RFDAF-Net produces more compact and distinct clustering for each category, with larger inter-class margins and smaller intra-class variances. This indicates that the model learns highly discriminative representations that effectively separate different disease categories while maintaining consistency within each class. In contrast, the baseline Swin-B model shows overlapping clusters and more dispersed feature distributions, particularly for visually similar categories, reflecting its limited ability to capture fine-grained discriminative features.

### 4.6 Analysis of the hyperparameter *α*

**Figure 9:**
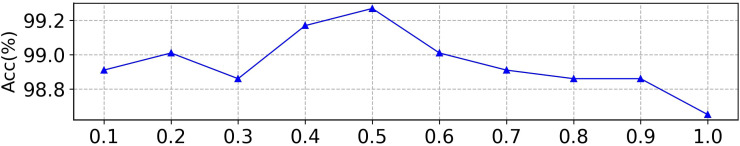
Influence of the hyperparameter *α*.

The hyperparameter *α* controls the degree of cross-branch feature suppression in the RFD module, specifically regulating how strongly salient regions detected in one branch are suppressed in the next. This encourages subsequent branches to focus on complementary regions and learn diverse features. As shown in **Figure 9**, model performance varies significantly with different values of *α*. Accuracy improves as *α* increases from 0.1, peaking at *α* = 0.5. Within this range, appropriate suppression effectively prevents feature redundancy across branches. Beyond *α* = 0.5, further increasing its value causes excessive suppression, which may remove useful information and reduce accuracy. These results confirm that controlled inter-branch suppression is essential for learning hierarchical and complementary features. The optimal value of *α* = 0.5 provides the best trade-off for maintaining feature diversity while minimizing redundancy.

### 4.7 Generalization analysis on other crops

To assess the robustness and transferability of RFDAF-Net beyond soybean crops, we extended our evaluation to the Paddy Disease Dataset (Petchiammal et al., 2023). This dataset comprises 10,407 high-resolution images collected from real-world paddy fields, categorized into one healthy class and nine disease classes (e.g., Blast, Dead Heart). Following standard protocols, the data was partitioned into a training set (7,808 samples, 75%) and a test set (2,599 samples, 25%). As shown in **Table 6**, RFDAF-Net achieved a superior accuracy of 99.15% on this dataset. It significantly outperforms the strong baseline Swin-B (97.45%) by 1.65% and surpasses the recent state-of-the-art model DIEC-ViT (98.94%). These results indicate that the proposed RFD and RFAF modules are not overfitted to specific soybean features. Instead, they demonstrate excellent generalization capabilities, effectively capturing discriminative pathological patterns across different plant species and complex agricultural environments.

**Table 6:** Comparison results with state-of-the-art models on the paddy disease dataset.

Model	Acc (%)
PiT-B (Heo et al., 2021)	97.26
PVT-B (Wang et al., 2021)	97.40
ViT-B (Dosovitskiy, 2020)	97.30
Swin-B (Liu et al., 2021)	97.45
ViT-B + EFG (Chang et al., 2024)	95.00
DIEC-ViT (Lin et al., 2025)	98.94
RFDAF-Net (Ours)	99.15

## 5 Limitations

While RFDAF-Net demonstrates state-of-the-art performance, it is essential to address several practical challenges regarding its deployment in real-world agricultural environments, particularly concerning computational feasibility. To provide a transparent analysis of the model’s complexity, we compared the parameter count of our proposed method against the baseline. As shown in **Table 7**, introducing the RFD and RFAF modules increases the total number of parameters from 86.74 M (Baseline Swin-B) to 105.01 M. While this additional complexity contributes to the performance gains, it inevitably raises computational costs. In practical agricultural environments, disease diagnosis often relies on resource-constrained edge devices, such as drones or handheld smartphones, which typically have limited memory and processing power. The current model size implies higher inference latency and energy consumption, potentially restricting its feasibility for real-time, large-scale field surveying tasks without hardware acceleration.

**Table 7:** Comparison of the number of parameters.

Model	Param (M)
Baseline (Swin-B)	86.74
RFDAF-Net (Swin-B)	105.01

In light of these findings, our future research will prioritize bridging the gap between high-performance algorithms and practical utility. Specifically, we aim to investigate model compression techniques, such as knowledge distillation and network pruning, to develop lightweight variants of RFDAF-Net suitable for edge deployment on drones or mobile devices. Furthermore, to mitigate the reliance on extensive expert annotations, we plan to explore semi-supervised or few-shot learning strategies, thereby enhancing the model’s adaptability to diverse and evolving agricultural scenarios with minimal data requirements.

## 6 Conclusions

In this study, we proposed the RFDAF-Net to address the critical challenges of feature redundancy and insufficient feature granularity in multi-scale learning for soybean disease recognition under complex field conditions. The introduced RFD module effectively disentangles multi-scale features by enhancing discriminative patterns and suppressing redundant information through a dual-pathway mechanism, enabling explicit capture of fine-grained details in shallow layers and high-level semantics in deeper layers. Furthermore, the RFAF module dynamically integrates these decoupled features using content-aware spatial weighting, achieving adaptive multi-scale fusion that significantly enhances representational capacity. Extensive experiments demonstrated that RFDAF-Net consistently outperforms state-of-the-art models across multiple backbone architectures and evaluation metrics. Ablation studies confirmed the individual contributions of both proposed modules, while visualization results using Grad-CAM, feature maps, and t-SNE provided interpretable evidence of the model’s ability to learn hierarchical and discriminative features. The analysis of key hyperparameters further validated the robustness and generalizability of the proposed approach.

## Data Availability

The original contributions presented in the study are included in the article/supplementary material. Further inquiries can be directed to the corresponding author.
